# Mammalian Target of Rapamycin Inhibition in *Trypanosoma cruzi*-Infected Macrophages Leads to an Intracellular Profile That Is Detrimental for Infection

**DOI:** 10.3389/fimmu.2018.00313

**Published:** 2018-02-20

**Authors:** Jorge David Rojas Márquez, Yamile Ana, Ruth Eliana Baigorrí, Cinthia Carolina Stempin, Fabio Marcelo Cerban

**Affiliations:** ^1^Facultad de Ciencias Químicas, Universidad Nacional de Córdoba (UNC), Centro de Investigaciones en Bioquímica Clínica e Inmunología (CIBICI), Consejo Nacional de Investigaciones Científicas y Técnicas (CONICET), Córdoba, Argentina

**Keywords:** mammalian target of rapamycin, macrophage, *Trypanosoma cruzi*, reactive oxygen species, NLRP3

## Abstract

The causative agent of Chagas’ disease, *Trypanosoma cruzi*, affects approximately 10 million people living mainly in Latin America, with macrophages being one of the first cellular actors confronting the invasion during *T. cruzi* infection and their function depending on their proper activation and polarization into distinct M1 and M2 subtypes. Macrophage polarization is thought to be regulated not only by cytokines and growth factors but also by environmental signals. The metabolic checkpoint kinase mammalian target of rapamycin (mTOR)-mediated sensing of environmental and metabolic cues influences macrophage polarization in a complex and as of yet incompletely understood manner. Here, we studied the role of the mTOR pathway in macrophages during *T. cruzi* infection. We demonstrated that the parasite activated mTOR, which was beneficial for its replication since inhibition of mTOR in macrophages by different inhibitors decreased parasite replication. Moreover, in rapamycin pretreated and infected macrophages, we observed a decreased arginase activity and expression, reduced IL-10 and increased interleukin-12 production, compared to control infected macrophages treated with DMSO. Surprisingly, we also found a reduced iNOS activity and expression in these macrophages. Therefore, we investigated possible alternative mechanisms involved in controlling parasite replication in rapamycin pretreated and infected macrophages. Although, cytoplasmic ROS and the enzyme indoleamine 2, 3-dioxygenase (IDO) were not involved, we observed a significant increase in IL-6, TNF-α, and IL-1β production. Taking into account that IL-1β is produced by activation of the cytoplasmic receptor NLRP3, which is one of the main components of the inflammasome, we evaluated NLRP3 expression during mTOR inhibition and *T. cruzi* infection. We observed that rapamycin-pretreated and infected macrophages showed a significant increase in NLRP3 expression and produced higher levels of mitochondrial ROS (mtROS) compared with control cells. Moreover, inhibition of mtROS production partially reversed the effect of rapamycin on parasite replication, with there being a significant increase in parasite load in rapamycin pretreated and infected macrophages from NLRP3 KO mice compared to wild-type control cells. Our findings strongly suggest that mTOR inhibition during *T. cruzi* infection induces NLRP3 inflammasome activation and mtROS production, resulting in an inflammatory-like macrophage profile that controls *T. cruzi* replication.

## Introduction

*Trypanosoma cruzi*, which is an intracellular protozoan parasite, is the etiologic agent of Chagas disease. This is a chronic condition affecting in the region of 10 million people worldwide who mostly reside in Latin America (1). Although, many of the mechanisms involved in the pathogenesis of Chagas disease are not well understood, the development and severity of American trypanosomiasis depends on immune-mediated mechanisms. The pathogen-associated molecular patterns (PAMPs) present in *T. cruzi* can be recognized by pattern recognition receptors, which is a crucial event in host resistance (2), with the capability of *T. cruzi* to infect and replicate within of different cells, among them macrophages (3), being an critical feature in its cycle (4).

Macrophages are key effector cells that participate in different stages of immune responses, such as antigen presentation, phagocytosis, and the secretion of bioactive molecules. Macrophages may either suppress *T. cruzi* replication or afford a favorable environment where it can reproduce and be distributed to other area within the body (3–6). Furthermore, macrophages are indispensible in tissue homeostasis and have a significant effect on protective immunity and pathological immune-mediated damage (7–9). Macrophages are generally thought to represent a range of activated phenotypes instead of stable subpopulations. Normally, these are separated into two specific phenotypes, classically activated macrophages (M1) and alternatively activated ones (M2) (10–14). The M1 types are considered effector cells when responding to microbial products or interferon-gamma (IFN-γ), and are characterized by a large antigen presenting ability and yielding pro-inflammatory cytokines, including interleukin-12 (IL-12), tumor necrosis factor alpha (TNF-α), nitric oxide (NO), and reactive oxygen species (ROS) (13, 15, 16). Consequently, M1 macrophages stimulate a polarized type I immune response that mediates host defense against infections of bacteria, viruses, and protozoa as well as tumor cells. On the other hand, M2 macrophages are induced by IL-13, IL-4, glucocorticoids, and IL-10, and they display an anti-inflammatory function, as well as promoting adaptive Th2 immunity and regulating angiogenesis, wound healing, and tissue remodeling (11–13).

Despite it being well known that the transcriptional response triggered by PAMP recognition of the surrounding microenvironments (including cytokines and growth factors) determines the phenotype and function of macrophages (13, 15–17), the intrinsic molecular mechanisms driving macrophage polarization are not yet been fully understood. Related to this, macrophage polarization is also thought to be regulated by environmental signals with the metabolic checkpoint kinase mammalian target of rapamycin (mTOR) mediating the sensing of the environmental and metabolic cues influencing macrophage polarization in a complex but still incompletely understood manner (18).

The mTOR protein is a conserved serine–threonine kinase which is known to influence multiple cellular functions, such as cell growth, proliferation, and survival by integrating signals from nutrients, energy status, growth factors, cytokines, and TLRs (19, 20). These signals are recognized by the PI3-K–Akt axis, and depending on the context, can activate mTORC1 or mTORC2 complexes (20). Immediately downstream of Akt is the tuberous sclerosis complex (TSC), which consists of the TSC tumor suppressors TSC1 and TSC2, and also Tre2-TBC1D7. These control the Ras homolog enriched in the brain (RHEB), which is a crucial GTPase regulator of mTORC1. On this complex being phosphorylated by Akt or ERK1/2, it becomes inhibited and RHEB is activated, resulting in the activation of mTORC1 (20). In a general context, protein synthesis is the best characterized process controlled by mTORC1, with this kinase directly phosphorylating the translational regulator eukaryotic translation initiation factor 4E (eIF4E)-binding protein1 (4EBP1) and S6 kinase (S6K), which in turn, promote protein synthesis (21).

The activation of mTOR has been shown to downregulate IL-12p70 and IL-23 production in LPS-stimulated human macrophages (22). Futhermore, IL-12 was enhanced, but IL-10 was blocked, following mTOR inhibition in mouse bone marrow-derived macrophages (BMDMs) and myeloid dendritic cells (23). These results suggest that mTOR activation might limit the pro-inflammatory responses. However, other studies have demonstrated that TSC1-deficient macrophages produce certain pro-inflammatory cytokines, including TNF-α, IL-6, and IL-12p40 as a response to multiple TLR ligands (18, 19, 23). Related to this, TSC1-deficient BMDMs treated with LPS led to an increased pro-inflammatory cytokine production, such as IL-6 and TNF-α, but showed a reduction in anti-inflammatory cytokine IL-10 secretion (17, 24, 25). Despite the increasing number of investigations examining the effects of mTOR on the immune system, little emphasis has been placed on the function of this pathway in macrophages during infection.

Inflammasomes are nucleotide-binding oligomerization domain like receptors (NLRs) expressed on macrophages and activated in *T. cruzi* infection. When the inflammasome complex is activated, the pro-IL-1β is cleaved in an IL-1β active form (26, 27). In this study, we investigated the role of mTOR on macrophage polarization and parasite growth during *T. cruzi* infection. We demonstrated that *T. cruzi* can activate mTOR, with this molecule being important for its survival since mTOR inhibition decreased the parasite load in macrophages. We also showed that mTOR inhibition in *T. cruzi*-infected macrophages activates NLRP3 inflammasome, upregulates IL-12, IL-6, TNF-α, IL-1β, and mitochondrial ROS (mtROS), but downregulates IL-10 and NO production as well as reducing arginase and iNOS activity and expression. These results indicate that mTOR activation induced by the parasite may be important in inducing a M2 phenotype which favors parasite replication. In contrast, mTOR inhibition in *T. cruzi*-infected macrophages induces an inflammatory M1-like phenotype which was able to limit parasite replication.

## Materials and Methods

### Mice

The BALB/c mice used were obtained from the Comisión Nacional de Energía Atómica (Buenos Aires, Argentina). Male TNFα-RKO (B6.129-Tnfrsf1a^tm1Mak^), IL6KO (B6.129S2-Il6/^Jtm1Copf^), and NLRP3 KO (B6.129S6-Nlrp3/J^tm1Bhk^) mice were purchased from the Jackson Laboratory. Male C57BL/6J mice were from Universidad Nacional de La Plata (Argentina). All mice were inbred and housed according to institutional guidelines (28).

### *Trypanosoma cruzi* Infection

The *T. cruzi* infection protocols were performed as described (29). Briefly, the infection was maintained through intraperitoneal inoculations every 11 days. Then, blood-derived trypomastigotes were used to infect monolayers of Vero cells. After 7 days, supernatants were collected and stored at −80°C.

### BMDMs and Peritoneal Macrophages (PM)

To obtain the BMDMs, the femur and tibia bones from different mice strains were flushed with cold RPMI 1640 containing 40 µg/mL gentamycin, following standard procedures (30). These recovered bone marrow progenitor cells were cultured in 100-mm bacteriologic plastic Petri dishes containing RPMI1640 supplemented with 10% FBS, 40 µg/mL gentamycin, 2 mM l-glutamine, and 30% L929-cell-conditioned medium (31) for 4 days. Then, bone marrow progenitors were supplemented with RPMI-LCM 30%. At day 6, BMDM were used as mentioned below, with a flow cytometric analysis of BMDM (Figure S1 in Supplementary Material) revealing that these cells were CD11b+ and F4/80+.

BALB/c mice underwent intraperitoneal infection as described previously (29), then PM were extracted and processed using several techniques. Non-infected animals were processed in parallel as control.

### Chemical Reagents

DMSO, LY294002, diphenyleneiodonium chloride (DPI), 1-methyl-dl-tryptophan, ATP, Griess reagent assay, DAPI, and phosphatase inhibitor (PhosphoStop) were obtained from Sigma-Aldrich (St. Louis, MO, USA); rapamycin and Fluorsave were purchased from Calbiochem (Darmstadt, Germany) and PP242 from Cayman Chemical (Ann Arbor, MI, USA); Recombinant mouse IFNγ, anti-F4/80+ (PE) and anti-CD11b+ (APC) FACS antibodies, recombinant mouse IL-4, ELISA Kits Assay IL-10, IL-12p70, TNFα, IL-6, and IL-1β were obtained from BioLegend (San Diego, CA, USA); LPS (from *E. coli* 0111:B4 strain) was from InvivoGen with DAF-FM, H2DCFDA, and MitoSOX TM Red probes and protein ladders being obtained from Thermo Fisher Scientifics (Waltham, MA, USA). Western blot (WB) antibodies anti-phospho-mTOR, anti-phospho-p70S6K, anti-phospho-4EBP1, anti-ATP-cytrate lyase, anti-β-actin, anti-NLRP3, and anti-IL-1β were acquired from Cell Signaling Technology (Danvers, MA, USA) and anti-Arginase I and anti-iNOS antibodies were obtained from Santa Cruz Biotechnology (Palo Alto, CA, USA). The Odyssey antibodies IRDye^®^ 680RD Donkey anti-Rabbit and IRDye^®^ 680RD Donkey anti-Mouse IgG were obtained from Li-Cor Biosciences (Lincoln, NE, USA). Finally, antibody anti-IgGh was from Biocientifica and protease inhibitor cocktail from Roche (Basilea, Switzerland).

### Inhibitors and Stimulus Treatments

Bone marrow-derived macrophage from Balb/c or C57BL6 mice at day 6 of differentiation were cultured for 3 h in RPMI-2%FBS for their adhesion. After that, cells were pretreated with DMSO as control; or pretreated with different inhibitors: 1-methyl-dl-tryptophan (1-MT, 100 µM, during 24 h, 37°C); diphenyleneiodonium chloride (DPI, 20 µM, during 3 h, 37°C), PP242 (40 or 80 nM, for 90 min., 37°C), LY294002 (10 or 50 nM, for 90 min.), rapamycin (50 or 100 nM, for 90 min.). After pretreatment, the BMDM were washed three times and then infected with *T. cruzi* (1:5, cell:parasites ratio). In addition, BMDM without inhibitor pretreatment were stimulated with LPS (1 µg/mL, as positive control of mTOR activation), LPS (1 µg/mL) + IFNγ (100 ng/mL), as positive control of M1 polarization, IL-4 (80 ng/mL), as positive control of M2 polarization, LPS (1 µg/mL) + ATP (5 mM) positive control of inflammasome. Then, cells and supernatants were collected for performing different techniques at 1, 3, 6, 12, 24, 48, and 72 p.i. The number of cells used for the different techniques was: 6 × 10^6^ cells/well for WB and ELISA, 3 × 10^6^ cells for Flow Cytometry, and 3 × 10^5^ cells for Immunofluorescence.

### Flow Cytometry

For the assessment of intracellular NO, cytoplasmic ROS (cROS), and mtROS, BMDM from each experimental condition were collected at 4°C and washed with PBS 2% FBS. First, cells were stained with APC labeled anti-CD11b and with FITC labeled anti-F4/80 for 20 min at 4°C. After that, BMDM were incubated with 20 µM DAF-FM diacetate probe for 30 min at 37°C for NO detection; 20 µM H2DCFDA probe for 20 min at 37°C for cROS detection; or 5 µM of a MitoSOX TM Red probe 20 min at 37°C for mtROS detection. Finally, these cells were analyzed by FACS as described previously (29).

### Cytokine Determination

IL-10, IL-12p70, TNFα, IL-6, and IL-1β were measured in culture supernatants by ELISA sandwich following the manufacturer’s guidelines.

### Western Blot

Cells were processed as previously described (29). Lysates were prepared by protein measurement using the Bradford micro-technique (32). Membranes were incubated overnight at 4°C with primary rabbit and mouse monoclonal antibodies anti-phospho-mTOR, anti-phospho-p70S6K, anti-phospho-4EBP1, anti-Arginase-I, anti-iNOS, anti-NLRP3, anti-IL-1β, anti-β-actin, or anti-ATP-cytrate lyase. Then, sheets were incubated with antibodies anti-Rabbit (IRDye^®^ 680RD Donkey anti-Rabbit) and anti-Mouse (IRDye^®^ 680RD Donkey anti-Mouse IgG) for 1 h, at room temperature and in darkness. An Odyssey CLx Infrared Imaging System (LI-COR, Inc.) was used to detect the bands.

### Immunofluorescence

Bone marrow-derived macrophages were treated as described in the Section “Inhibitors and Stimulus Treatments,” and non-internalized parasites were eliminated by performing washes with RPMI 24 h later. Parasite growth in BMDM was determined by counting the number of intracellular amastigotes using immunofluorescence assays as described (33). For nuclear staining, coverslips were incubated with DAPI, before being washed and incubated in mounting media FluorSave overnight. The slides were observed using an Olympus BX41 microscope (Olympus Corporation, Tokyo, Japan) and a Leica DMi8 microscope (Leica Microsystems). Images were processed with ImageJ software.

### Griess Assay

Nitric oxide levels were obtained by measuring those of the stable end product (nitrites) with Griess reagent assay (34). Absorbance at 540 nm was measured by the Bio Rad microplate reader and optical density was converted to micro molar of nitrites using a standard curve of sodium nitrite.

### Arginase Reaction

Arginase activity was measured in macrophage lysates as previously described (33, 35, 36). Cells were lysed with 100 µL of 1% Triton X-100 containing protease inhibitor cocktail and phosphatase inhibitor. Then after 30 min, lysates were prepared for protein measurement by using the Bradford micro-technique (32). The urea (produced by arginine hidrolysis) was measured at 540 nm. The results were expressed as micrograms of urea per microgram of protein.

### Statistics

Statistical analyses were performed using the Student’s *t*-test of GraphPad Prism software version 6.0 (GraphPad Software). Discrepancies with a value of *p* < 0.05 were considered significant.

## Results

### *T. cruzi* Infection Induces mTOR Activation in Macrophages

**Figure 1:**
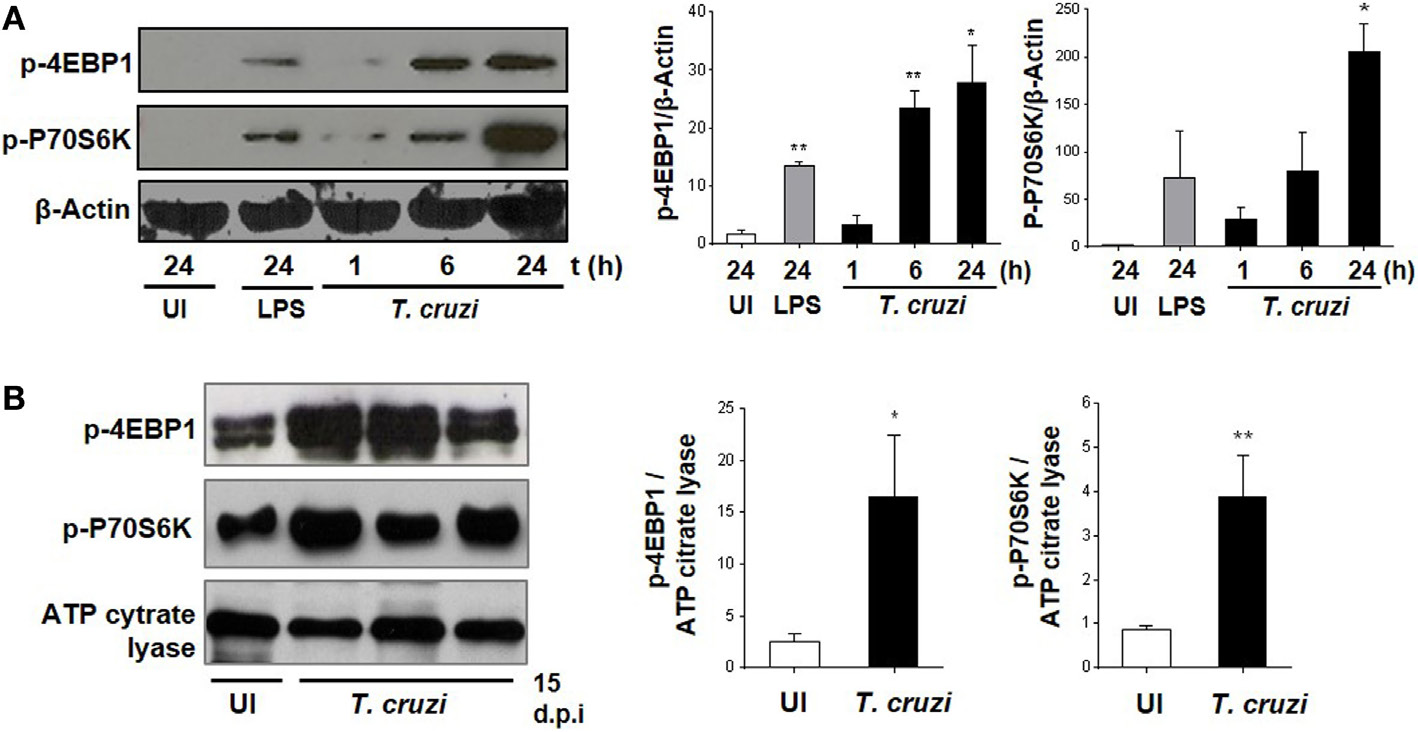
*Trypanosoma cruzi* infection induces mammalian target of rapamycin (mTOR) activation in macrophages. mTOR activation was determined by phosphorylation of mTORC1 substrates, p-4EBP1, and p-P70S6K using Western blot assays (WB). **(A)** Bone marrow-derived macrophage (BMDM) cultured in RPMI without stimulus and without infection were used as control (UI), and BMDM stimulated with LPS 1 µg/mL were used as positive control (LPS). BMDM were infected by adding *T. cruzi* trypomastigotes (1:5, cell:parasite ratio). Infected BMDM were obtained and analyzed at different times (1, 6, and 24 h). Left panel shows a representative experiment and right panel shown densitometry analysis using ImageJ software. Bars represent mean ± SD from three independent experiments. Protein loading was evaluated by β-Actin expression, *p < 0.05 and **p < 0.005 vs. UI. **(B)** Peritoneal macrophages (PM) from *T. cruzi*-infected Balb/c mice were obtained at 15 days postinfection (d.p.i.) and PM from uninfected Balb/c mice were used as control (UI). Left panel shows a representative experiment and right panel shows densitometry analysis using ImageJ software. Bars represent mean ± SD of three independent experiments. The protein loading was evaluated by ATP citrate lyase expression (*p < 0.05 and **p < 0.005 vs. UI).

It has been previously demonstrated that the invasion of trypomastigotes is reduced in HeLa cells treated with the mTOR inhibitor rapamycin (37). However, this has not yet been explored for mTOR function in macrophages during *T. cruzi* infection (38). Therefore, we infected BMDM with *T. cruzi* trypomastigotes and evaluated mTOR activation by WB through phosphorylation of its substrates 4EBP1 and P70S6K. The mTOR activation was observed at 6 and 24 h postinfection (Figure 1A), and in addition, we evaluated mTOR activation in peritoneal cells from mice at 15 days postinfection. The peritoneal cells from infected mice revealed an increase in 4EBP1 and P70S6K phosphorylation compared to control cells from uninfected mice (Figure 1B), with these results indicating that mTOR is an important pathway induced by the parasite in macrophages.

### mTOR Activation in Macrophages Is Essential for Parasite Replication

**Figure 2:**
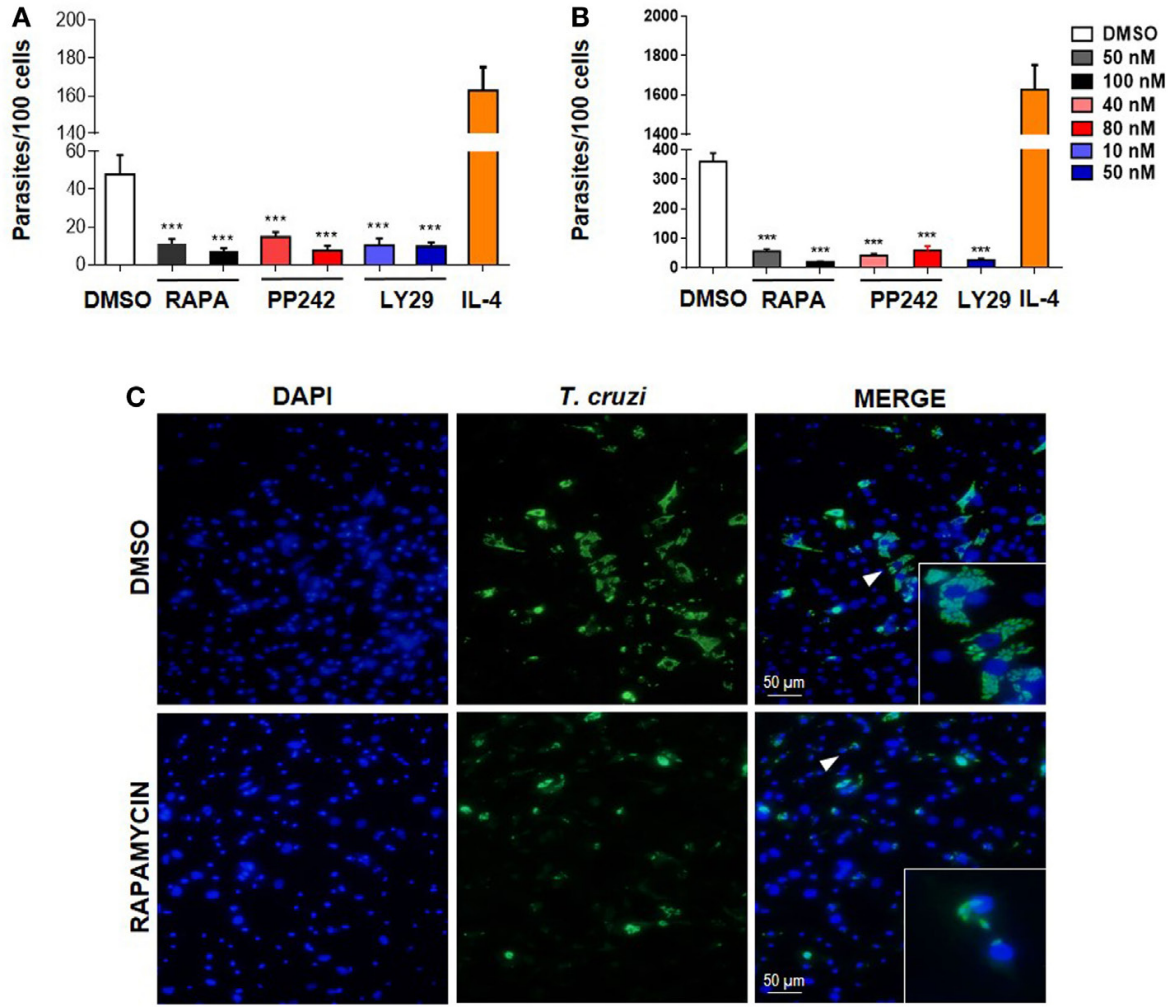
Mammalian target of rapamycin (mTOR) activation in macrophages is essential for parasite replication. **(A)** Bone marrow-derived macrophage (BMDM) and **(B)** peritoneal macrophages (PM) from Balb/c mice were pretreated with DMSO as control or with different mTOR inhibitors: rapamycin (50 or 100 nM), PP242 (40 or 80 nM), and LY294002 (10 or 50 nM) during 90 min. Cells were washed and then were infected with *Trypanosoma cruzi* trypomastigotes (1:5, cell:parasite ratio) during 24 h. Besides, BMDM (A) or PM (B) without inhibitors pretreatment were stimulated with IL-4 (80 ng/mL) and infected with *T. cruzi* trypomastigotes (1:5, cell:parasite ratio) during 24 h as positive control of infection. After that, non-internalized parasites were removed and 72 h later intracellular amastigotes were counted by indirect immunofluorescence. Parasite replication was expressed as number of parasites per 100 cells, quantified by ImageJ software. Bars represent mean ± SD from three independent experiments (***p < 0.001 vs. DMSO). **(C)** A representative image from DMSO or rapamycin pretreated BMDM shown cell nucleus stained with DAPI and parasites in green. Inserts show an area from the image (arrowhead) at higher magnification, indicating infected macrophages.

To determine if mTOR activation is important for parasite survival, we performed experiments by targeting this pathway at different levels. First, BMDM were treated with the mTOR inhibitors rapamycin or PP242, or with Ly249002 (a PI3K inhibitor) or DMSO as control and incubated 90 min. Then, cells were washed and infected with *T. cruzi* trypomastigotes. The number of intracellular parasites was evaluated 72 h later by immunofluorescence. It was observed that pretreatment with rapamycin, PP242, and Ly294002 significantly reduced the number of parasites in BMDM (Figures 2A,C) and also in peritoneal infected cells compared to DMSO-treated and infected cells (Figure 2B). However, the number of parasites increased in IL-4 stimulated and infected cells, used as positive control of parasite replication (Figures 2A,B).

None of these treatments produced any cytotoxic effect on the cells according to cell viability measured by the release of LDH in the supernatants of these cultures (data not shown). The inhibitory effect of the drugs was not a consequence of its action on the trypomastigotes, since inhibitors were removed before incubation with parasites. In addition, it has been reported that pretreatment of parasites with rapamycin does not alter their infectivity in HeLa cells, even for high drug concentrations (37). Thus, our results indicate that the *T. cruzi* parasite activates the mTOR pathway in macrophages in order to promote its survival, since mTOR inhibitors control parasite replication.

### Modulation of *T. cruzi*-Induced Macrophage Polarization by Rapamycin

**Figure 3:**
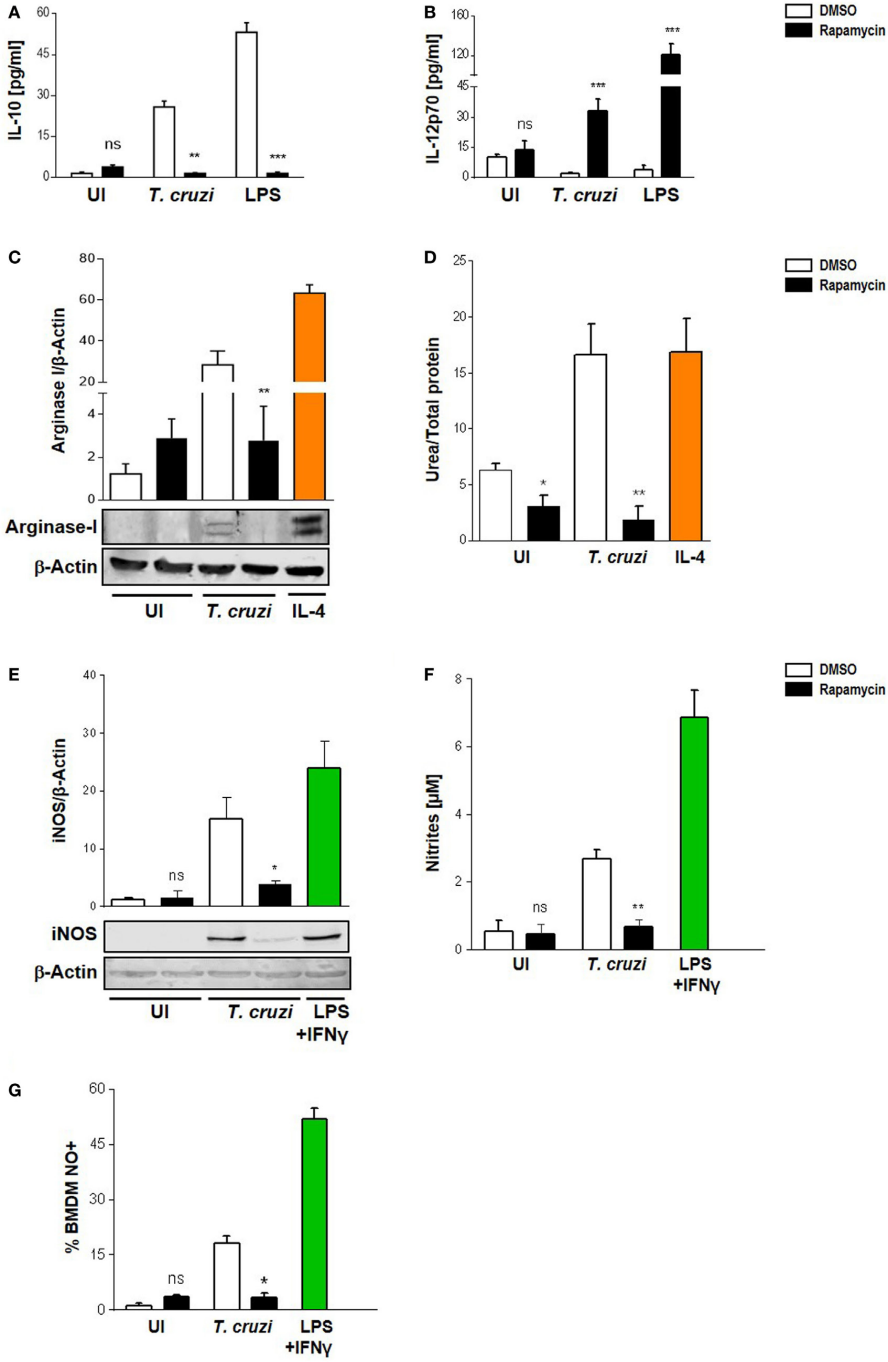
Modulation of *Trypanosoma cruzi*-induced macrophage polarization by rapamycin. Macrophage polarization was evaluated through cytokine production, Arginase I, and iNOS expression and activity. Bone marrow-derived macrophage (BMDM) from Balb/c mice were pretreated with DMSO as control, or with rapamycin (100 nM) during 90 min. After pretreatment cells were washed and uninfected (UI) or *T. cruzi*-infected (*T. cruzi*) (1:5, cell:parasite ratio) BMDM were cultured during 24 h and then processed for the different experiment. **(A)** IL-10 and **(B)** IL-12p70 production were measured by ELISA sandwich-assays. Besides, BMDM with or without rapamycin pretreatment were stimulated with LPS 1 µg/mL as positive control (LPS) during 24 h. Arginase I **(C)** and iNOS **(E)** expression from cellular lysates were performed by western blot at 24 h postinfection, using as loading control β-actin. Besides, BMDM were stimulated with IL-4 (80 ng/mL) or LPS (1 µg/mL) + IFNγ (100 ng/mL) during 24 h as positive controls for Arginase-I (C) or iNOS (E) expression, respectively. Upper panel shows densitometry analysis using ImageJ software. Bars represent mean ± SD from three independent experiments (***p < 0.001 vs. DMSO). Bottom panel shows a representative experiment. **(D)** Arginase activity was determined by urea production assay 24 h p.i. **(F)** iNOS activity was evaluate by Griess reaction on supernatants 24 h p.i. **(G)** Besides, iNOS activity was determined by FACS. BMDM were stained with anti-F480 (PE) and anti-CD11b (APC) and then incubated with DAF-FM-DA probe (20 µM, FITC), 30 min at 37°C for intracellular NO detection. Bars represent mean ± SD from three independent experiments (*p < 0.05, **p < 0.005, and ***p < 0.001 vs. DMSO; ns, no significant difference vs. DMSO).

It has been suggested that mTOR in macrophages enhances the expression of M2-associated cytokines (38–40). Moreover, the pharmacological inhibition of mTOR with rapamycin resulted in an inhibition of LPS induction of IL-10 mRNA and protein, but enhanced the pro-inflammatory cytokine TNFα production (41). Thus, to determine the role of mTOR in *T. cruzi*-induced cytokines and whether this could influence the survival of the parasite in macrophages, we performed experiments by incubating BMDM with rapamycin or DMSO as control for 90 min, then, cells were washed and infected with *T. cruzi* trypomastigotes. On evaluating IL-10 and IL-12 production 24 h later by ELISA, it was found that rapamycin pretreatment induced a switch in cytokine production in infected macrophages. Moreover, a rise in IL-12 but a reduction in IL-10 production were observed in rapamycin-pretreated and infected macrophages compared to DMSO control macrophages (Figures 3A,B). To achieve a better understanding about the mechanisms implicated in controlling the parasite in rapamycin-treated macrophages, we evaluated Arginase I and iNOS, which are hallmarks of the M1 or M2 activation profiles, respectively. BMDM were pretreated for 90 min with rapamycin or DMSO as control, after which, the cells were washed and infected with *T. cruzi* trypomastigotes and Arginase I and iNOS expression and activities were measured 24 h later. Arginase was slightly induced by the parasite whereas rapamycin pretreatment reduced its expression and activity (Figures 3C,D), correlating with a decreased IL-10 production (Figure 3A). Surprisingly iNOS expression was also reduced in rapamycin pretreated and infected macrophages (Figure 3E), as well as the nitrite production in culture supernatants (Figure 3F) and the frequency of NO producing cells (Figure 3G).

### Cytoplasmic ROS Production and IDO Activity Are Not Involved in Parasite Control in Rapamycin-Treated and Infected Macrophages

**Figure 4:**
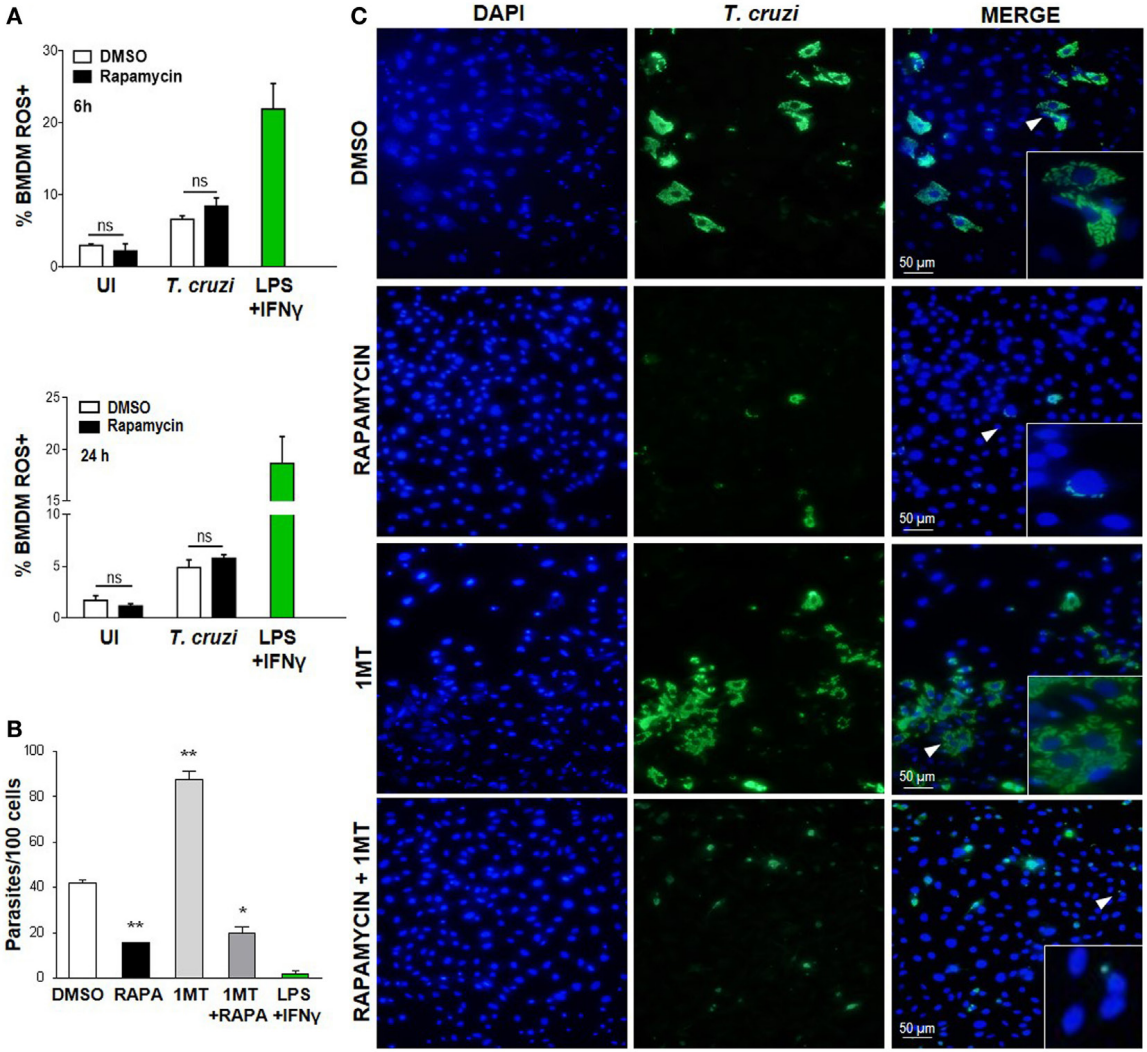
Decreased *Trypanosoma cruzi* replication by mammalian target of rapamycin inhibition was independent of cytoplasmic ROS and IDO. **(A)** Bone marrow-derived macrophages (BMDM) from C57BL/6 mice were pretreated with DMSO as control or with rapamycin (100 nM) during 90 min. After pretreatment cells were washed and uninfected (UI) or *T. cruzi*-infected (*T. cruzi*) (1:5, cell:parasite ratio) were cultured at different times and then processed for the experiments. Besides, BMDM were stimulated with LPS + IFNγ (1 µg/mL + 100 ng/mL), during 24 h as positive control. BMDM at 6 and 24 h postinfection were stained with anti-F480 (PE) and anti-CD11b (APC) mAbs. Then, cells were incubated with 20 µM H2DCFDA probe, 20 min at 37°C for intracellular reactive oxygen species (ROS) detection. Bars represent mean ± SD of from three independent experiments (ns, no significant difference vs. DMSO). **(B–C)** Parasite replication in BMDM from C57BL6 mice pretreated with DMSO as control, or with Rapamycin (RAPA: 100 nM) during 90 min, or with 1-methyl tryptophan (1-MT: 100 µM; 24 h) or with 1-MT (24 h) + RAPA (90 min). After pretreatment BMDM were washed and then infected with *T. cruzi* trypomastigotes (1:5, cell:parasite ratio) during 24 h. Besides, BMDM without inhibitors pretreatment, were stimulated with LPS + IFNγ (1 µg/mL + 100 ng/mL), during 24 h as positive control. After that, non-internalized parasites were removed and 72 h later intracellular amastigotes were counted by indirect immunofluorescence. **(B)** Intracellular replication of *T. cruzi* is expressed as number of parasites per 100 cells, quantified by ImageJ software and represent mean ± SD from three independent experiments (*p < 0.05 and **p < 0.005, vs. DMSO). **(C)** A representative image shows cell nucleus stained with DAPI and parasites in green. Inserts show an area from the image (arrowhead) at higher magnification, indicating infected macrophages.

It has been previously shown that the ROS resulting from the respiratory burst have an important role in *T. cruzi* control (42–44), but ROS may be involved in cellular signaling and proliferation of this parasite (45). Nevertheless, on evaluating cROS production in rapamycin-treated and infected macrophages at different points after infection by FACS (using H2DCFDA, which measures cROS and mainly detects H_2_O_2_), we did not observe any differences in the frequency of the cROS producing macrophages between rapamycin-treated and infected macrophages and DMSO-treated and infected macrophages at 6 or 24 h p.i. (Figure 4A) or 24 h p.i. (Figure 4B). However macrophages stimulated with LPS plus IFN-γ, used as a positive control of ROS production, revealed a high frequency of cROS producing cells (Figure 4A).

On the other hand, the enzyme indoleamine 2,3-dioxigenase (IDO) of the tryptophan catabolism is involved in inhibiting intracellular pathogen replication (46, 47); moreover, IDO activity is induced by inflammatory cytokines and is essential for limiting the parasite’s reproduction in macrophages since *T. cruzi* amastigotes are sensitive to the l-kynurenine downstream metabolites (48). In order to examine the effect of IDO activity on the regulation of parasite growth *in vitro* in our experimental system, BMDM were cultured in the presence or absence of 1-methyl-d-tryptophan (1-MT), an IDO inhibitor, for 24 h, before being treated with rapamycin for 90 min and then infected with *T. cruzi*. After 24 h, the non-internalized parasites were eliminated through washes, and the intracellular parasites were counted by immunofluorescence 72 h later. IDO blockade with 1-MT was demonstrated to cause a strong stimulatory effect on intracellular parasite growth, as previously reported (48). However, 1-MT treatment did not reverse the effect of rapamycin (Figures 4B,C), indicating that IDO is not involved in limiting parasite growth in rapamycin-pretreated and infected macrophages.

### mTOR Inhibition Alters the Cytokine Balance of Macrophages toward a Pro-inflammatory Phenotype upon *T. cruzi* Infection

**Figure 5:**
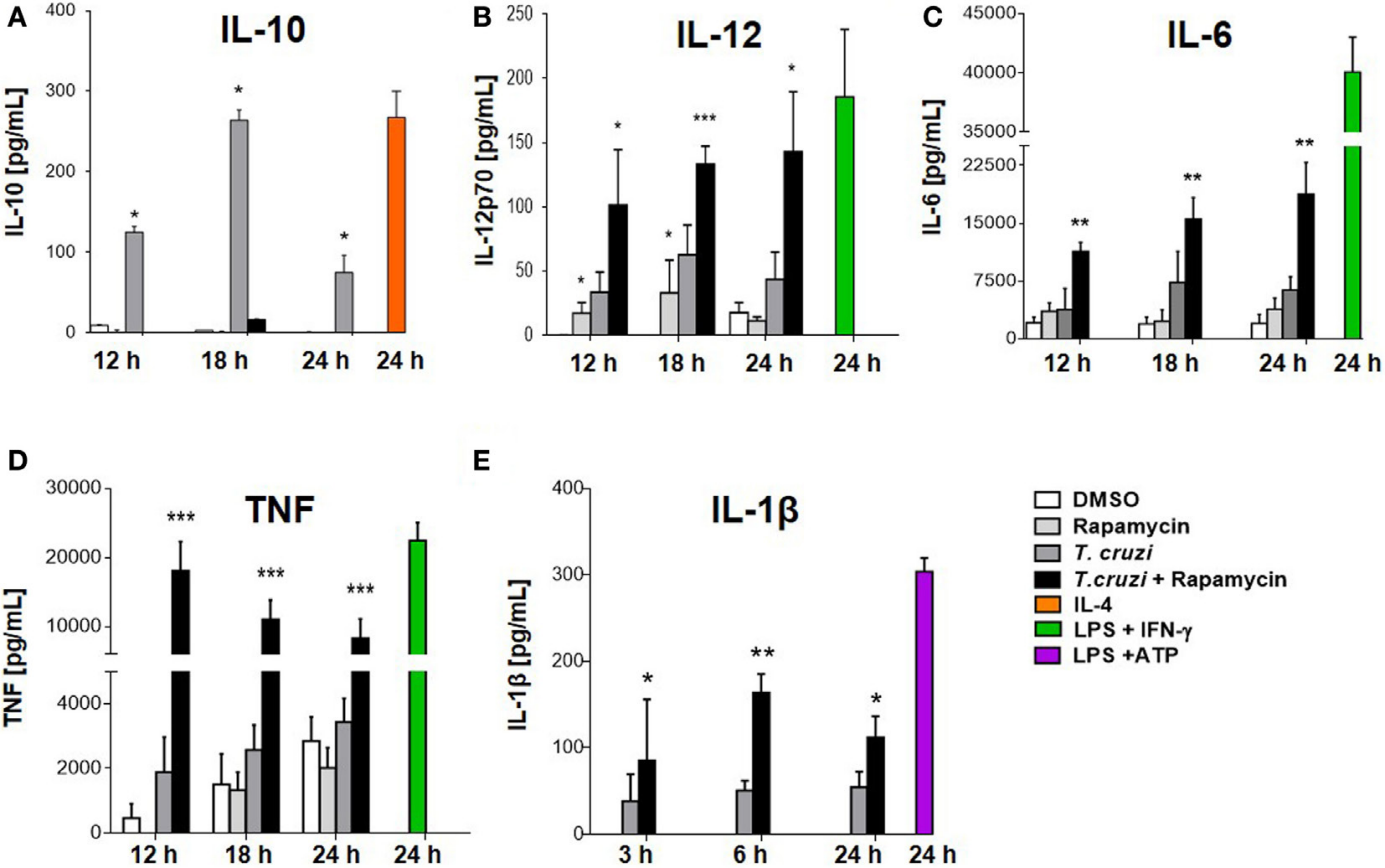
Mammalian target of rapamycin inhibition alters cytokine balance in macrophages toward a pro-inflammatory phenotype upon *Trypanosoma cruzi* infection. Bone marrow-derived macrophages (BMDMs) from C57BL/6 mice were pretreated with DMSO as control or with rapamycin (100 nM) during 90 min. After pretreatment, BMDM uninfected or infected with *T. cruzi* trypomastigotes (1:5, cell:parasite ratio) were cultured at different times. At 12, 18, and 24 h postinfection, supernatants were collected and processed to determine the IL-10 **(A)**, IL-12p70 **(B)**, IL-6 **(C)**, TNFα **(D)**, and IL-1β **(E)** production by ELISA Sandwich. Besides, supernatants from BMDM stimulated with IL-4 (80 ng/mL), or with LPS (1 µg/mL) + IFNγ (100 ng/mL), or with LPS (1 µg/mL) + ATP (5 mM) during 24 h were used as positive controls. Bars panels represent mean ± SD from three independent assays (*p < 0.05, **p < 0.005, and ***p < 0.001 vs. DMSO).

We have shown that rapamycin pretreatment induced an increase in IL-12 and a corresponding reduction in IL-10 production in infected macrophages (Figures 3A,B). It has been reported that rapamycin differentially modulates both pro- and anti-inflammatory cytokine production in macrophages in response to LPS such as TNF-α, IL-6, and IL-1β, among others (24, 25, 49). BMDM were pretreated for 90 min with rapamycin or DMSO as control. Then, cells were washed and infected with *T. cruzi* trypomastigotes and cytokine production was evaluated at different time points after infection. mTOR inhibition by rapamycin in BMDM infected with *T. cruzi* led to a strong upregulation of IL-12 production (Figures 3B and 5B). At the same time, the anti-inflammatory cytokine IL-10 was remarkably suppressed (Figures 3A and 5A), whereas, importantly, mTOR inhibition increased the production of the pro-inflammatory cytokines IL-6, TNF-α, and IL-1β (Figures 5C–E respectively). These results indicate that mTOR inhibition alters the cytokine balance of macrophages toward a pro-inflammatory phenotype upon *T. cruzi* infection.

### Rapamycin Induces NLRP3 Expression in *T. cruzi*-Infected Macrophages, Which Is Relevant in the Control of Parasite Replication

**Figure 6:**
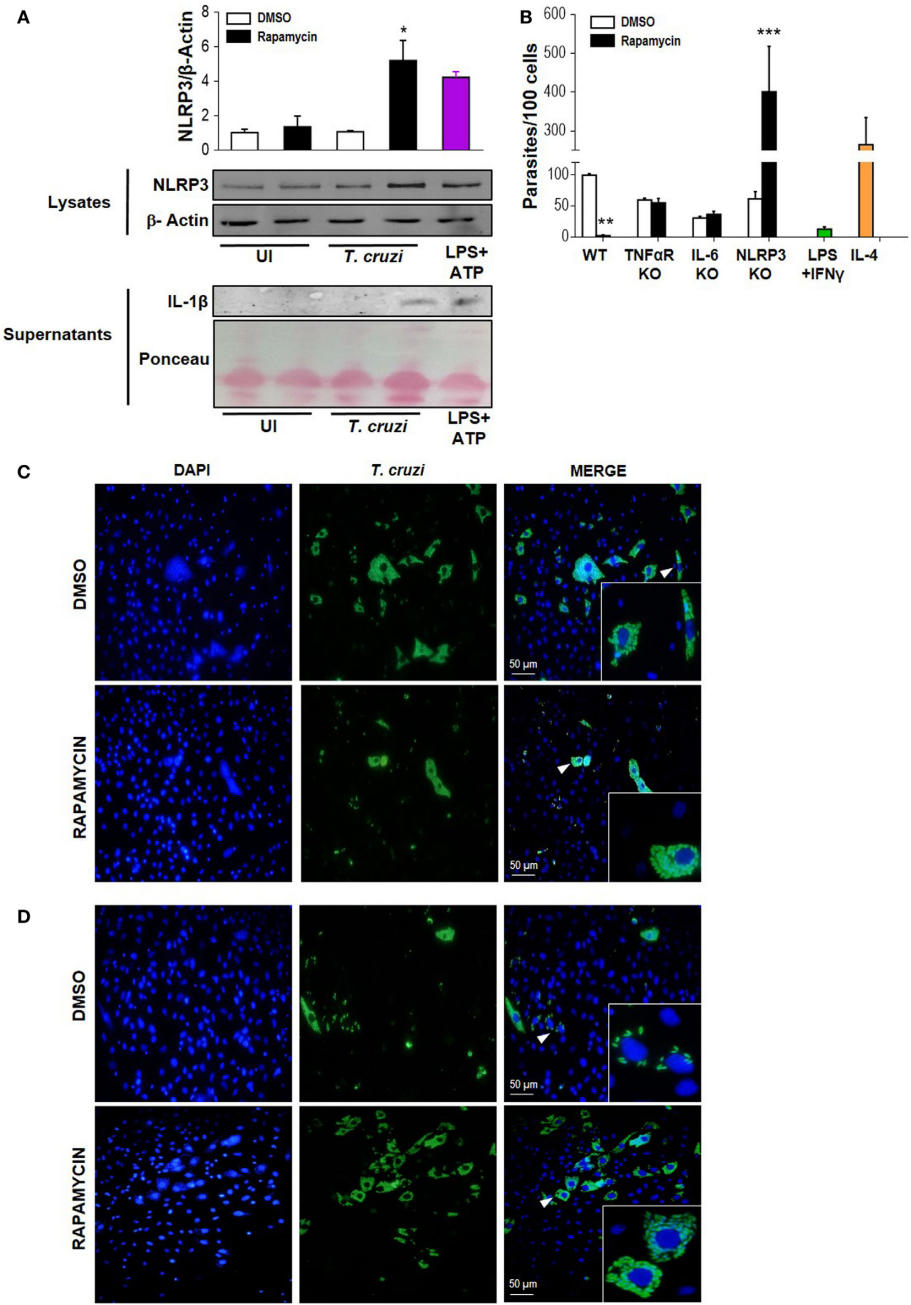
Rapamycin induces NLRP3 activation in *Trypanosoma cruzi*-infected macrophages, a relevant event to control parasite replication. **(A)** NLRP3 activation was determined by NLRP3 expression (upper panel) and cleaved IL-1β release (bottom panel) using western blot assays (WB). Bone marrow-derived macrophages (BMDMs) from C57BL/6 mice were pretreated with DMSO as control or with rapamycin (100 nM) during 90 min. After rapamycin pretreatment cells were washed and uninfected (UI) or *T. cruzi*-infected BMDM (*T. cruzi*) (1:5, cell:parasite ratio) were cultured during 6 h. Besides, BMDM were stimulated with LPS (1 µg/mL) + ATP (5 mM) as positive control. Then, cell lysates and supernatants were processed for WB. A representative experiment of three independent experiments is shown. The protein loading for NLRP3 and IL-1β was evaluated by β-Actin expression and by Ponceau staining, respectively. Densitometry analysis from cell lysates was performed by ImageJ software (*p < 0.05 and **p < 0.005 vs. DMSO). **(B)** Parasite replication in BMDM from wild-type (WT), TNFαR KO, IL-6 KO, and NLRP3 KO mice. BMDM were pretreated with DMSO as control or with rapamycin (100 nM) during 90 min. After pretreatment, cells were washed and infected with *T. cruzi* trypomastigotes (1:5, cell:parasite ratio) during 24 h. Besides, WT BMDM without rapamycin pretreatment were stimulated with LPS (1 µg/mL) + IFNγ (100 ng/mL) and infected with *T. cruzi* trypomastigotes (1:5, cell:parasite ratio) during 24 h as negative control of parasite replication. On the other hand, WT BMDM without rapamycin pretreatment were stimulated with IL-4 (80 ng/mL) and infected with *T. cruzi* trypomastigotes (1:5, cell:parasite ratio) during 24 h as positive control of parasite replication. After that, non-internalized parasite were removed, and 72 h later, intracellular amastigotes were counted by indirect immunofluorescence. Intracellular replication of *T. cruzi* is expressed as number of parasites per 100 cells, quantified by ImageJ software and represent mean ± SD from three independent experiments (*p < 0.05, **p < 0.005, and ***p < 0.001 vs. DMSO). A representative image from WT **(C)** and NLRP3 KO **(D)** BMDM show cell nucleus stained with DAPI and parasites in green. Insert show an area from the image (arrowhead) at higher magnification, indicating infected macrophages.

It has been shown that NLRP3 is involved in the antiparasitic response against *T. cruzi*, but the mechanisms involved are known to depend on the experimental design employed (50). Here, we evaluated NLRP3 expression and IL-1β production in rapamycin pretreated and infected macrophages. Briefly, BMDM were pretreated for 90 min with rapamycin or DMSO as control and cells were washed and infected with *T. cruzi* trypomastigotes. Cell lysates and supernatants were collected 6 h p.i., and NLRP3 expression and IL-1β production were measured by wild type. We observed that mTOR inhibition by rapamycin in *T. cruzi*-infected BMDM led to a potent upregulation of NLRP3 and an increase in IL-lβ production (Figure 6A), with BMDM stimulated with ATP plus LPS serving as a positive control of inflammasome activation.

Then, to study the relevance of NLRP3 activation during mTOR inhibition in infected macrophages, we obtained BMDM from NLRP3 KO or wild-type (WT) mice and pretreated them with rapamycin or DMSO for 90 min. Then, cells were infected and *T. cruzi* intracellular replication was measured by immunofluorescence. In agreement with Figure 2 and Figure S2 in Supplementary Material, a reduction in parasite growth in rapamycin pretreated and infected BMDM from WT was observed. However, rapamycin pretreated and infected BMDM from NLRP3 KO showed a strong increase in parasite replication, which was even more robust than that observed in IL-4 stimulated BMDM used as control (Figure 6B). Representative images from DMSO or rapamycin treated and infected WT BMDM (Figures 6C) and NLRP3 KO BMDM are shown in Figure 6D. In addition, in rapamycin pretreated and infected BMDM from TNFα-R KO and IL-6 KO mice there was an increase in parasite replication compared with rapamycin-pretreated and infected BMDM from WT mice. However, this effect was less evident than in the BMDM from NLRP3 KO mice (Figure 6B and Figure S3 in Supplementary Material).

### mtROS Production Is Important in Controlling Parasite Replication during mTOR Inhibition in *T. cruzi*-Infected Macrophages

**Figure 7:**
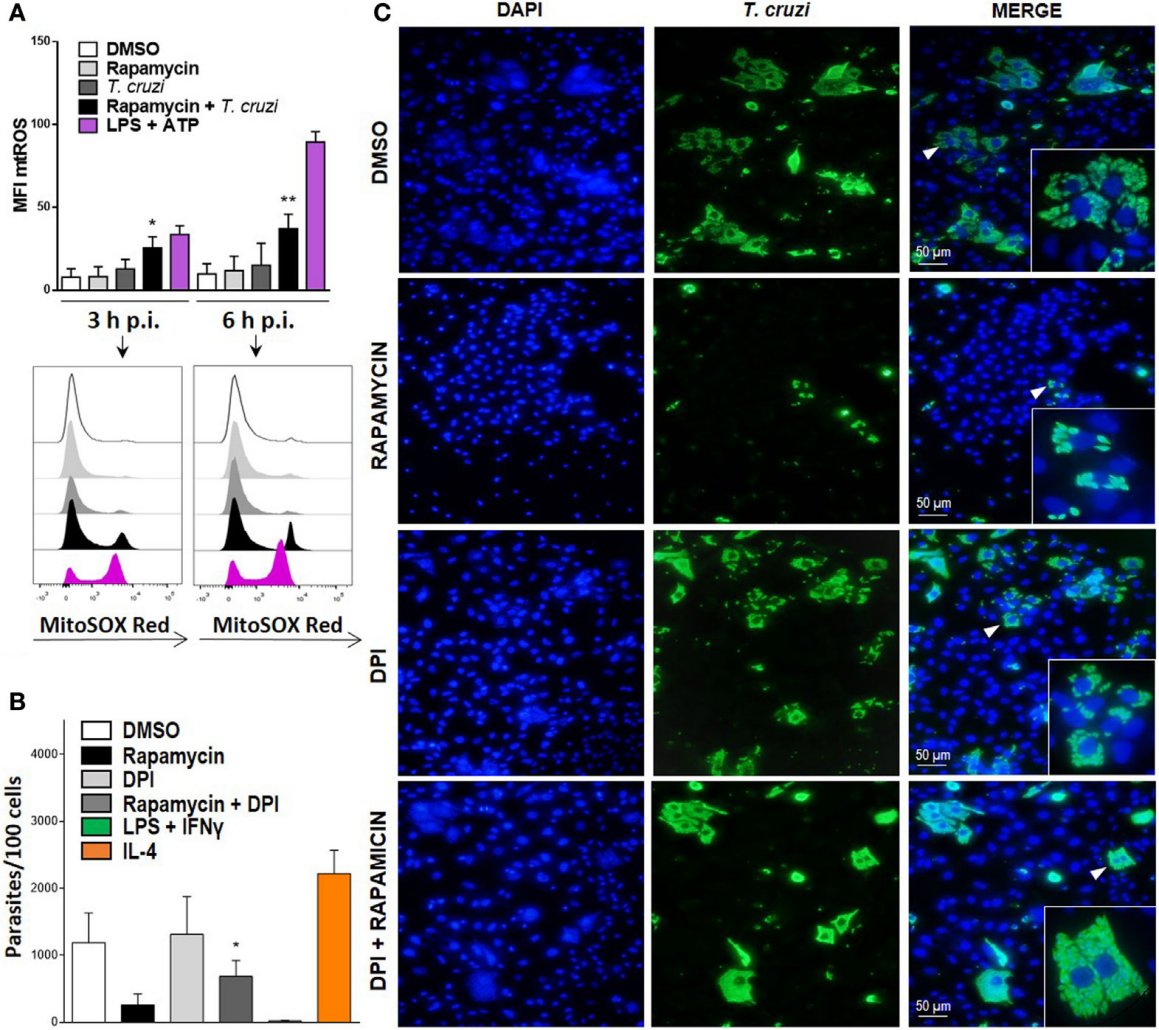
Mitochondrial ROS (mtROS) production is important to control parasite replication during mammalian target of rapamycin (mTOR) inhibition in *Trypanosoma cruzi*-infected macrophages. (**A)** Bone marrow-derived macrophage (BMDM) from C57BL/6 mice pretreated with DMSO as control or with rapamycin (100 nM) during 90 min. After pretreatment cells were washed and uninfected or infected *T. cruzi* BMDM (1:5, cell:parasite ratio) were cultured at 3 and 6 h. Besides, BMDM without rapamycin pretreatment were stimulated with LPS + ATP (1 µg/mL and 5 mM) during 3 and 6 h. At indicates times postinfection (p.i.), BMDM were stained with anti-F480 (FITC) and anti-CD11b (APC) mAbs. Then, cells were incubated with 5 µM MitoSOX probe (PE) 15 min at 37°C and analyzed by Flow cytometry. Bars display mean fluorescence intensity (MFI) of mtROS on F4/80+ CD11b+ gated populations. Experiments were repeated three times with similar results being obtained and are expressed as mean ± SD (*p < 0.05 and **p < 0.005 vs. DMSO). A representative histogram of at least three independent experiments is shown. **(B)** Parasite replication in BMDM from C57BL6 mice pretreated with DMSO as control (24 h) rapamycin (100 nM, 90 min), with DPI (20 µm, 3 h) or with DPI + rapamycin. After pretreatment, cells were washed and infected with *T. cruzi* trypomastigotes (1:5, cell:parasite ratio) during 24 h. Besides, BMDM without inhibitors pretreatment, were stimulated with LPS (1 µg/mL) + IFNγ (100 ng/mL) or with IL-4 (80 ng/mL) and infected with *T. cruzi* trypomastigotes (1:5, cell:parasite ratio) during 24 h as controls. After that, non-internalized parasite were removed and 72 h later intracellular amastigotes were counted by indirect immunofluorescence. Intracellular replication of *T. cruzi* is expressed as number of parasites per 100 cells, quantified by ImageJ software and represent mean ± SD from three independent experiments (*p < 0.05; vs. rapamycin). **(C)** A representative image from DMSO, rapamycin, DPI, rapamycin + DPI pretreated BMDM, show cell nucleus stained with DAPI and parasites in green. Inserts show an area from the image (arrowhead) at higher magnification, indicating infected macrophages.

It has been previously shown that mitochondria participate in inflammasome activation (51). The NLRP3 inflammasome is the best characterized among various inflammasome complexes, with its activation depending on different stress signals, including ROS production promoted by mitochondrial dysfunction (51). Thus, we evaluated mtROS production in rapamycin treated and infected BMDM at different points after infection using MitoSOX, which measures mtROS and mainly detects superoxide radical. We found a significant increase in mtROS production in rapamycin-pretreated and infected BMDM, with increased levels of mtROS being observed in ATP plus LPS-stimulated BMDM, used as positive control (Figure 7A). Then, to evaluate whether mtROS is relevant for controlling parasite replication, BMDM from WT mice were pretreated with rapamycin, DPI (NADPH oxidase inhibitor), rapamycin + DPI, or DMSO before being infected, and the parasite load was studied by immunofluorescence. It was observed that BMDM incubated with rapamycin plus DPI had a significantly higher parasite load compared to rapamycin-pretreated BMDM (Figure 7B), with a representative image from DMSO, rapamycin, DPI, and rapamycin plus DPI treated and infected BMDM being shown in Figure 7C. Additionally, we have observed that DPI inhibits mtROS production induced by rapamycin pretreatment and *T. cruzi* infection, Figure S4 in Supplementary Material. This may indicate that mtROS would participate in the control of *T. cruzi* replication. These findings strongly suggest that mTOR inhibition during *T. cruzi* infection in BMDM induces inflammasome NLRP3 activation and mtROS production, which controls parasite survival.

## Discussion

In order to control the *T. cruzi* infection, it is necessary that cytokine-mediated macrophage activation leads to intracellular killing of the parasite. Moreover, M1 polarization is closely related to parasite removal, whereas M2 polarization can be effective in countering the development of an oxidative and inflammatory pathology in Chagas disease (35, 36, 52).

As the protein mTOR is a critical regulator of the host cell metabolism, it is a logical target to be manipulated by invasive pathogens such as *T. cruzi*. In this investigation, we examined the role of the mTOR pathway in macrophage polarization induced by *T. cruzi* and we observed that mTOR is activated *in vitro* by the parasite in BMDM. In addition, peritoneal cells obtained from infected mice showed an increase in mTOR activation compared to those from uninfected mice. These results clearly indicate that mTOR is an important pathway induced by the parasite in macrophages considering that its inhibition during infection shifts these macrophages to an M1-like inflammatory profile by reducing IL-10 and arginase activity and expression. Rapamycin pretreated and infected macrophages revealed an activation of the NLRP3 inflammasome and an increased production of IL-12, IL-6, TNF-α, IL-1β, and mtROS. Thus, these macrophages have an increased ability to limit parasite replication, which was clearly demonstrated using three different inhibitors of the mTOR pathway (rapamycin, PP242, and LY249002).

The selective deletion of signals through mTORC1 in macrophages promotes M1 cytokines (53), whereas deletion of signals through mTORC2 inhibits the generation of M2 while maintaining intact the generation of M1 (54). In TLR-induced pro-inflammatory cytokine production, the role of the TSC-mTOR pathway is still unclear. It was reported that in TSC2-deficient MEFs, the pro-inflammatory responses are reduced as a result of impaired IKK activation and NF-κB translation to the nuclei (54). Nevertheless, other studies have found that rapamycin treatment enhances the IL-12 production in myeloid DCs by stimulating NF-κB activation, but prevents IL-12 production in bone marrow-derived DCs and monocyte-derived ones (40, 55). Moreover, TSC1-deficient macrophages produce elevated pro-inflammatory cytokines in response to an LPS stimulation, such as TNF-α, IL-12, and IL-6 (18, 19, 24). The reason for this apparent inconsistency is still unknown, but possibly originates from the different cell types or the length of mTOR inhibition with rapamycin. Here, we found that mTOR inhibition by rapamycin (90 min) in BMDM infected with *T. cruzi* led to a potent upregulation of IL-12 production, whereas the anti-inflammatory cytokine IL-10 was notably suppressed. Furthermore, mTOR inhibition increased the production of the pro-inflammatory cytokines IL-6 and TNF-α. These results indicate that mTOR inhibition alters the cytokine balance of macrophages toward a pro-inflammatory phenotype upon *T. cruzi* infection.

The role of the mTOR pathway in the regulation of IL-1β expression has been examined in both mouse and human macrophages (38, 56). In addition, other authors have used TSC1-deficient macrophages and macrophage cell lines to study the involvement of TSC1 in pro-inflammatory cytokine IL-1β expression and investigated the associated molecular mechanism. They reported that at the level of both the mRNA and protein, the LPS-induced pro-IL-1β synthesis was significantly downregulated in TSC1-deficient macrophages, by extended rapamycin (48 h) treatment or mTOR deletion (18). In contrast, we found that mTOR inhibition by rapamycin (90 min) in *T. cruzi*-infected BMDM led to a potent upregulation of IL-1β production. However, it is possible that differences in exposure time of the macrophages to rapamycin, was crucial in determining the outcome.

Deletion of Akt1 promotes upregulation of inducible NO synthase and IL-12 (M1 activation) and enhances bacteria clearance (57, 58). We observed that pretreatment with mTOR inhibitors (rapamycin, PP242) and PI3K inhibitor (Ly294002) significantly reduced the number of parasites in BMDM and in peritoneal infected cells compared to DMSO-treated and infected cells. Also, we found that a short period of mTOR inhibition previous to infection induced an inflammatory profile in these macrophages similar to an M1 polarization without iNOS expression. It is also possible that mTOR inhibition also modifies *T. cruzi* invasion in BMDM, since this was demonstrated through the inhibition of PI3K (59, 60).

Importantly, macrophage alternative (or M2) activation is induced by signaling through the IL-4R *via* STAT6. Related to this, TSC1-deficient macrophages have been found to fail to alternatively activate in response to IL-4, reflecting the fact that pronounced mTORC1 signaling is a strong negative regulator of alternative activation (61, 62). This was not due to effects on STAT6 phosphorylation, but rather to feedback inhibition of Akt phosphorylation through effects on the insulin receptor substrate 2, which is also engaged by IL-4R signaling (18, 62). Interestingly, in TSC1-deficient macrophages, low-dose rapamycin (which preferentially inhibits mTORC1 but not mTORC2) allows an alternative activation in response to IL-4. However, recent work using higher concentrations of rapamycin (which also inhibits mTORC2), Torin (which inhibits both mTORC1 and mTORC2), and Rictor deficient macrophages has revealed that mTORC2 is critical for alternative activation (54, 63). Thus, mTORC2 acts in parallel with pSTAT6 to promote an alternative activation, in part by cooperating in events that lead to expression of IRF4 (63). In our present investigation, rapamycin induced not only a decrease in the activity and expression of arginase and IL-10 production in *T. cruzi*-infected BMDM but also produced a decrease in iNOS expression and activity. Moreover, although cROS was not induced, rapamycin induced mtROS and IL-1β production along with pro-inflammatory cytokines such as IL-12, IL-6, and TNF-α.

The NLRs are part of the response to *T. cruzi*, as first reported for NOD1 (27). Although KO animals for this receptor have a higher parasite burden and mortality than WT, they are still capable of producing cytokines at systemic level, suggesting that other NLRs may also contribute to resistance against this parasite. NLRP3 is activated in response to lysosomal damage generated by the escape of protozoan from the parasitophora vacuole, but this is independent of the K+ flow and ROS generation (26). These studies showed that NO production is eliminated in primary macrophages from NLRP3 KO *T. cruzi* Y strain-infected mice. However, some authors (64) argue that this phenomenon is dependent on both IL-1β and IL-1R, while others have postulated that this is independent of these molecules (50). Nevertheless, these results emphasize the complexity of the antiparasitic response orchestrated by the NLRP3 inflammasome together with other innate receptors and reflected in various microbicidal mechanisms assembled against *T. cruzi* (51, 65). In our study, rapamycin pretreatment in *T. cruzi*-infected BMDM induced NLRP3 expression, mtROS and IL-1β production, but not cROS or NO production.

Several studies have indicated that cROS is a necessary secondary messenger in order to achieve signaling caspase-1/ASC inflammasome activation (66, 67), but this was demonstrated for a feedback cycle of IL-1β signaling of cROS activation in *T. cruzi*-infected macrophages (50), with the molecular mechanisms involved in cROS production induced by IL-1β remaining to be clarified. In previous investigations, IL-1β was reported to promote phospholipase A2, thereby stimulating the release of arachidonic acid. However, since arachidonic acid is able to activate NADPH oxidase to afford superoxide, it is a possibility that this fatty acid serves as an intermediate in the IL-1β-induced activation of the enzymes, thus resulting in the production of ROS (67, 68). In our study, we demonstrated that rapamycin pretreatment in *T. cruzi*-infected macrophages induces mtROS production, which is involved in the control of *T. cruzi* replication, since an inhibitor of NADPH oxidase (DPI) partially reverses the effect of rapamycin. Certainly DPI is as potent as rotenone in inhibiting the production of superoxide and H_2_O_2_ by mitochondrial respiration.

It is important to emphasize that we cannot exclude the possible implication of mTOR inhibition in the limitation of some nutrients relevant for *T. cruzi* proliferation within BMDM, thus potentiating microbicidal effect of mtROS. This would be supported by the effect of partial inhibition produced by DPI treatment. In addition, it would be interesting to study the mechanism of mtROS inhibition by DPI and its effect on *T. cruzi* replication. It would be possible that changes in macrophages metabolism lead them to a metabolic program mediated by oxidative phosphorylation in detriment of glycolysis that is characteristic of M1 macrophages (69), since DPI influences mitochondrial respiration. However, the relevance of mtROS in controlling the intracellular growth of a microorganism has already been demonstrated in macrophages treated with metformin and infected with *M. tuberculosis*. In fact, metformin is an activator of the AMP-activated protein kinase, which has an important role in cellular energy homeostasis. Subsequently, our results indicate that the modulation of macrophage metabolism may be a new therapeutic tool in the control of intracellular infections (70).

In summary, the relevance of this study lies in the fact that treatment with rapamycin prior to macrophage *T. cruzi* infection controlled the intracellular growth of the parasite through inflammasome NLRP3 expression and the production of mtROS. In contrast, the iNOS and IDO enzymes were not involved. Thus, induction of mtROS may be relevant in the control of pathogen infections residing in macrophages.

## Ethics Statement

The Institutional Experimentation Animal Committee of the Chemical Sciences Faculty authorized the experimental protocols (no. 2016-213). This committee adopts the guidelines of the “Guide to the care and use of experimental animals” and those of the “Institutional Animal Care and Use Committee Guidebook.”

## Author Contributions

The authors point out their participation in the conception, design, drafting, and revision of the investigation, which provided an important intellectual content. The study was directed, reviewed, and approved by FC and CS. The authors have agreed to be accountable for all aspects of this work, in terms of integrity, accuracy and all other related issues.

## Conflict of Interest Statement

The authors declare that the research was conducted in the absence of any commercial or financial relationships that might be construed as a potential conflict of interest.
