# Supplementary material for: Mammalian Target of Rapamycin Inhibition in Trypanosoma cruzi-Infected Macrophages Leads to an Intracellular Profile That Is Detrimental for Infection
Source: Front Immunol. 2018 Feb 20;9:313. doi: 10.3389/fimmu.2018.00313 (PMC5826284; doi:10.3389/fimmu.2018.00313)
Supplement: Supplementary file 1 [file Image_1.PDF]

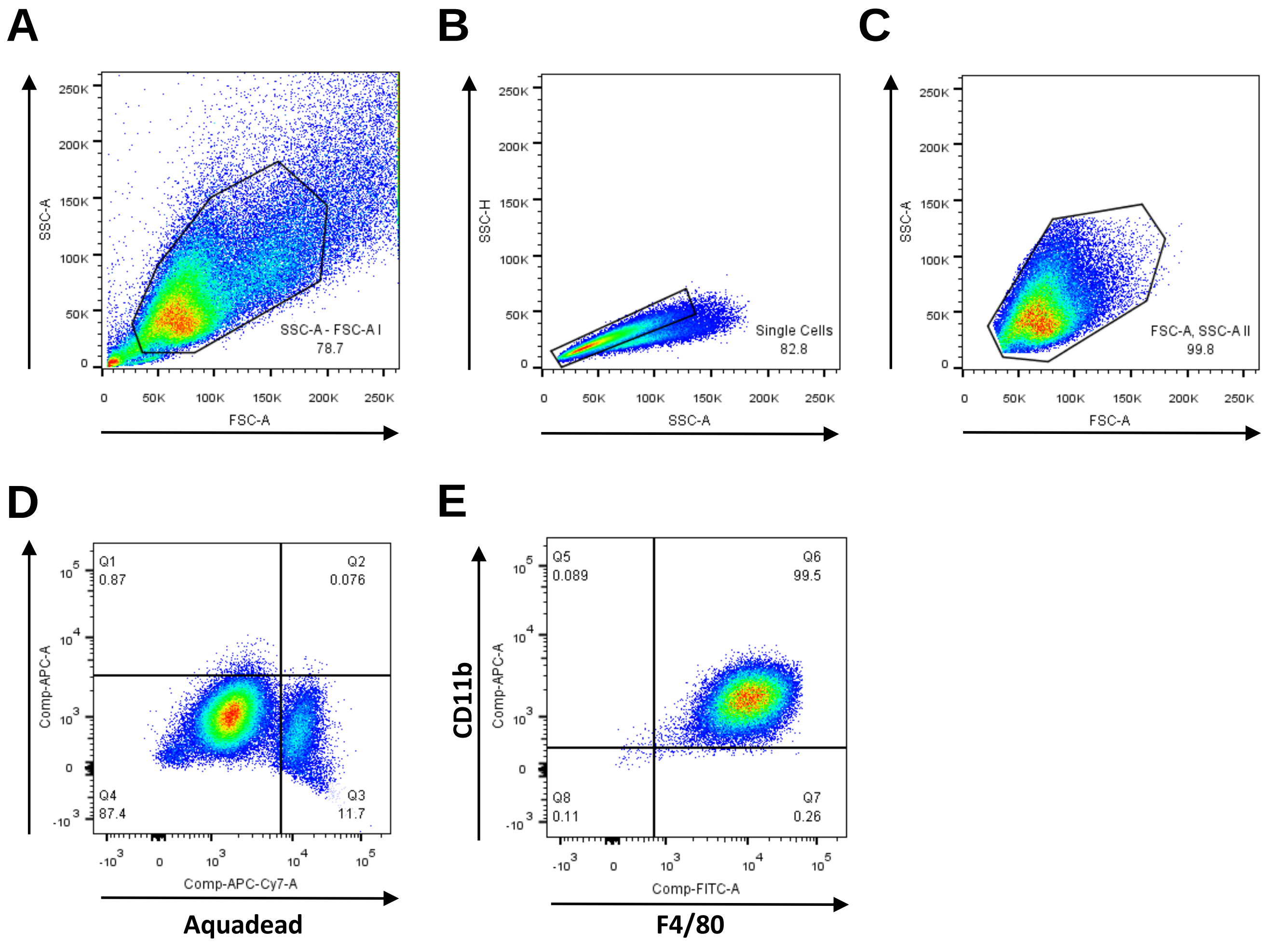

Figure S1. Gating strategy for BMDM FACS analysis assessed by FlowJo software. (A) FSC-SCC > **(B)** Single cells > **(C)** Single cells > **(D)** Live/ Dead Aqua stain > **(E)** F4/80+; CD11b+. BMDM from Balb/c and C57BL6 mice were collected and stained with aquadead (life/dead discrimination), anti-F480 (PE) and anti-CD11b (APC) mAbs for purity determination and different techniques.
