# Supplementary material for: Mammalian Target of Rapamycin Inhibition in Trypanosoma cruzi-Infected Macrophages Leads to an Intracellular Profile That Is Detrimental for Infection
Source: Front Immunol. 2018 Feb 20;9:313. doi: 10.3389/fimmu.2018.00313 (PMC5826284; doi:10.3389/fimmu.2018.00313)
Supplement: Supplementary file 2 [file Image_2.PDF]

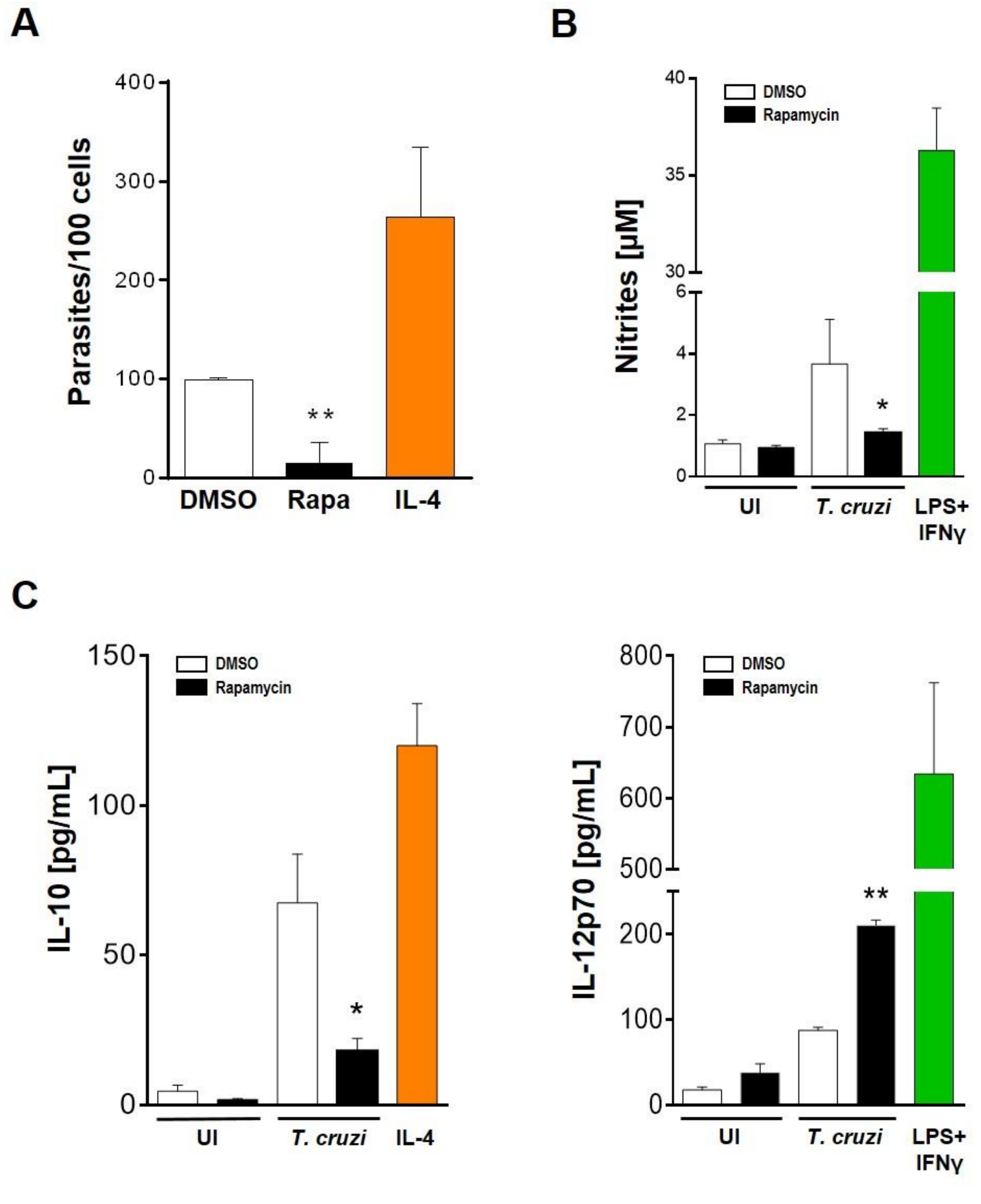

Figure S2. **Modulation of *T. cruzi*-induced macrophage polarization by Rapamycin is also observed in BMDM from C57BL/6 background. (A)** BMDM from C57BL/6 mice were pretreated with DMSO as control or with Rapamycin (100 nM) during 90 min. After pretreatment cells were washed and infected with *T. cruzi* trypomastigotes (1:5, cell:parasite ratio) cultured during 24 h. Besides, BMDM without inhibitors pretreatment were stimulated with IL-4 (80 ng/mL) during 24 h and infected with *T. cruzi* trypomastigotes (1:5, cell:parasite ratio) as positive control of infection (IL-4). After that, non internalized parasite were removed and 72 h later intracellular amastigotes were counted by indirect immunofluorescence. Parasite replication was expressed as number of parasites per 100 cells, quantified by ImageJ software. Bars represent mean ± SD of three independent experiments (***p<0.001 vs. c). **(B-C)** BMDM from C57BL/6 mice were pretreated with DMSO as control or with Rapamycin (100 nM) during 90 min. After pretreatment cells were washed and uninfected (UI) or infected with *T. cruzi* trypomastigotes (1:5, cell:parasite ratio) and cultured at different times. At 24 h p.i supernatants were collected and processed to determinate **(B)** iNOS activity by Griess reaction at 24 h p.i, and **(C)** the IL-10 and IL-12p70 production by ELISA Sandwich. Besides, supernatants from BMDM stimulated with IL-4 (80 ng/mL) or with LPS (1 μg/mL) + IFNγ (100 ng/mL) were used as controls. Bars panels represent mean ± SD of three independent experiments (*p< 0.05; **p<0.005; vs. DMSO).
