# Supplementary material for: Mammalian Target of Rapamycin Inhibition in Trypanosoma cruzi-Infected Macrophages Leads to an Intracellular Profile That Is Detrimental for Infection
Source: Front Immunol. 2018 Feb 20;9:313. doi: 10.3389/fimmu.2018.00313 (PMC5826284; doi:10.3389/fimmu.2018.00313)
Supplement: Supplementary file 3 [file Image_3.PDF]

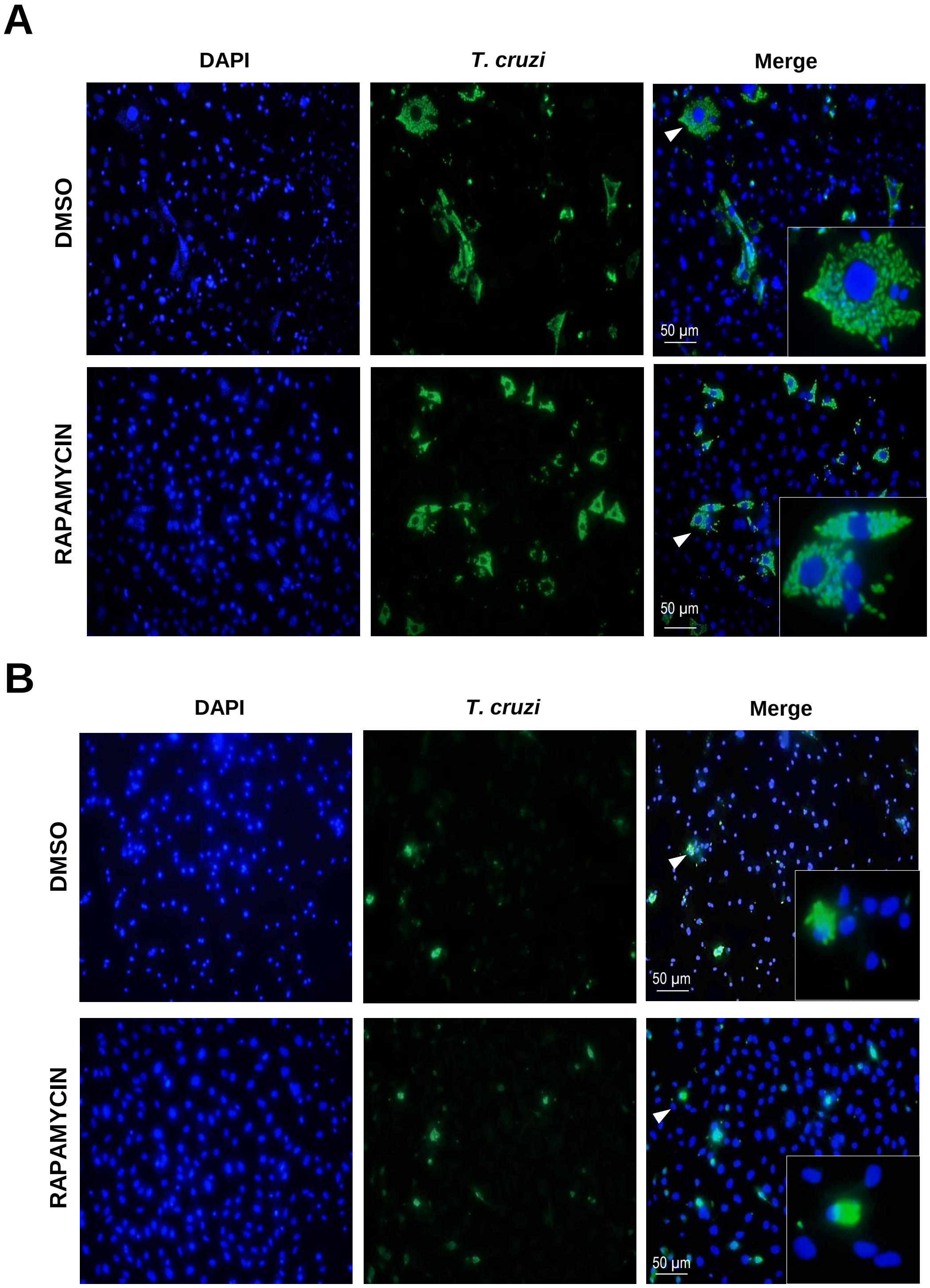

Figure S3. Parasite load in BMDM from TNFα-R KO and IL-6 KO pretreated with Rapamycin. Parasite replication in BMDM from TNF-R KO and IL-6 KO mice. BMDM were pretreated with DMSO as control or with Rapamycin (100 nM) during 90 min. After pretreatment BMDM were infected with *T. cruzi* trypomastigotes (1:5, cell:parasite ratio) during 24h. After that cells were washed and the number of intracellular parasite was analyzed by immunofluorescence 72 h later. A representative image from TNF-R KO **(A)** and IL-6 KO **(B)** BMDM, shown cell nucleus stained with DAPI and parasites in green. Inserts displayed an area at higher magnification (arrowhead) indicating infected macrophages.
