# Supplementary material for: Mammalian Target of Rapamycin Inhibition in Trypanosoma cruzi-Infected Macrophages Leads to an Intracellular Profile That Is Detrimental for Infection
Source: Front Immunol. 2018 Feb 20;9:313. doi: 10.3389/fimmu.2018.00313 (PMC5826284; doi:10.3389/fimmu.2018.00313)
Supplement: Supplementary file 4 [file Image_4.PDF]

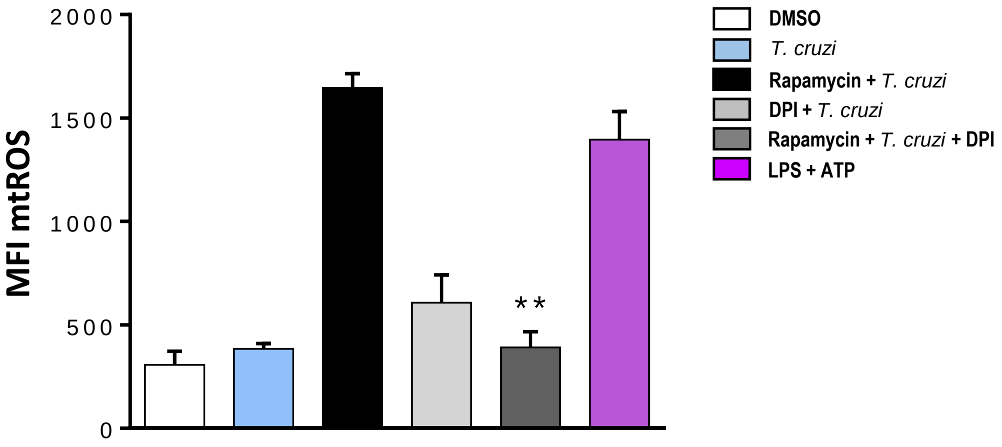

Figure S4. **mtROS production in presence of DPI.** BMDM from C57BL/6 mice pretreated with Rapamycin, (100 nM, 90 min.), DPI (20 µm; 3 h) or DPI (20 µm; 3 h) + Rapamycin (100 nM, 90 min.). Then cells were washed and infected with *T. cruzi* trypomastigotes (1:5, cell:parasite ratio). After that, uninfected (DMSO) or infected BMDM were cultured during 6 h. Besides, BMDM without inhibitor pretreatment were stimulated with LPS (1 µg/mL) + ATP (5 mM) during 6 h as positive control. At indicates times p.i. BMDM were stained with anti-F480 (FITC) and anti-CD11b (APC) mAbs. Then, cells were incubated with 5µM MitoSOX probe (PE) 15 min. at 37°C and analyzed Flow cytometry. Bars display mean fluorescence intensity (MFI) of mitochondrial ROS (mtROS) on F4/80+ CD11b+ gated populations (**$p<0.005$ vs. Rapamycin + *T. cruzi*).
